# P28 An analysis of the appropriateness of antimicrobial prescribing at a tertiary centre: reflections from the National Point Prevalence Survey

**DOI:** 10.1093/jacamr/dlae136.032

**Published:** 2024-08-23

**Authors:** Dylan Zeki Salih, Luke Bedford, David Enoch, Vanessa Wong, Susannah Burrows, Reem Santos, Fiona Cooke, Theodore Gouliouris

**Affiliations:** Sidney Sussex College, Cambridge, UK; Clinical Microbiology and Public Health Laboratory, University of Cambridge Hospitals NHS Foundation Trust, Cambridge, UK; Clinical Microbiology and Public Health Laboratory, University of Cambridge Hospitals NHS Foundation Trust, Cambridge, UK; Clinical Microbiology and Public Health Laboratory, University of Cambridge Hospitals NHS Foundation Trust, Cambridge, UK; Clinical Microbiology and Public Health Laboratory, University of Cambridge Hospitals NHS Foundation Trust, Cambridge, UK; Clinical Microbiology and Public Health Laboratory, University of Cambridge Hospitals NHS Foundation Trust, Cambridge, UK; Clinical Microbiology and Public Health Laboratory, University of Cambridge Hospitals NHS Foundation Trust, Cambridge, UK; Clinical Microbiology and Public Health Laboratory, University of Cambridge Hospitals NHS Foundation Trust, Cambridge, UK; Department of Medicine, University of Cambridge, Cambridge, UK

## Abstract

**Background:**

Between 18 September 2023 and 27 October 2023, Cambridge University Hospitals NHS Foundation Trust (CUH) partook in the UK Health Security Agency (UKHSA) National Point Prevalence Survey (PPS) of healthcare-associated infections, antimicrobial use and stewardship. This survey was the first to assess the appropriateness of individual antimicrobial prescriptions.

**Objectives:**

To use the data collected during the PPS to identify antimicrobial agents associated with inappropriate prescribing at CUH and investigate the factors contributing to this trend.

**Methods:**

Using the UKHSA PPS protocol, data pertaining to antimicrobial prescriptions at CUH during the survey period were collected. All prescriptions captured in the PPS with an associated appropriateness score were analysed. Grouping prescriptions by individual antimicrobial agent allowed identification of antimicrobial agents with a statistically significant proportion of ‘inappropriate’ prescriptions. Further analysis of the prescriptions of these agents, using the PPS data, permitted identification of factors which contributed to this association.

**Results:**

A total of 1064 patient reviews were conducted on 51 wards. Overall, 477/1064 (45%) of patients were on antimicrobials (mean number of antimicrobial prescriptions per patient=0.7, SD=0.7). Of the 52 agents used, co-amoxiclav (*n*=147, 19%), piperacillin/tazobactam (*n*=86, 11%), fluconazole (*n*=45, 6%) and sulfamethoxazole/trimethoprim (*n*=44, 6%) were the commonest. Appropriateness was assessed for 565 (79.5%) of the 711 antimicrobial prescriptions with 488 (86.4%) considered appropriate and 77 (13.6%) inappropriate. Co-amoxiclav was identified as the only antimicrobial agent with a statistically significant association with inappropriate prescribing (*P*<0.001, OR 2.9, 95% CI 1.7–4.8). Further analysis revealed that co-amoxiclav prescriptions for treatment were, relative to other antimicrobials, less likely to adhere to either national or local guidelines (*P*=0.006, OR 0.4, 95% CI 0.2–0.8) and significantly more likely to be prescribed to treat a condition which does not require antimicrobials (*P*=0.004, OR 0.3, 95% CI 0.1–0.7) and to treat an infection which required a broader spectrum agent (*P*<0.001, OR 0.2, 95% CI 0.1–0.5).

**Conclusions:**

Co-amoxiclav was most commonly associated with misuse/overuse and efforts to improve prescribing at CUH should focus on this agent. Further work is required to understand the factors that drive this association in order to improve prescribing.

